# Physical Interaction between HPV16E7 and the Actin-Binding Protein Gelsolin Regulates Epithelial-Mesenchymal Transition via HIPPO-YAP Axis

**DOI:** 10.3390/cancers13020353

**Published:** 2021-01-19

**Authors:** Paola Matarrese, Rosa Vona, Barbara Ascione, Marco G. Paggi, Anna Maria Mileo

**Affiliations:** 1 Center for Gender-Specific Medicine, Oncology Unit, Istituto Superiore di Sanità, 00161 Rome, Italy; paola.matarrese@iss.it (P.M.); rosa.vona@iss.it (R.V.); barbara.ascione@iss.it (B.A.); 2 Cellular Networks and Molecular Therapeutic Targets, Proteomics Unit, IRCCS—Regina Elena National Cancer Institute Rome, 00144 Rome, Italy; 3 Tumor Immunology and Immunotherapy Unit, IRCCS—Regina Elena National Cancer Institute Rome, 00144 Rome, Italy

**Keywords:** HPV16E7, EMT, HIPPO-YAP pathway, Gelsolin, actin cytoskeleton, RhoGTPases

## Abstract

**Simple Summary:**

Human papilloma viruses cause benign or malignant hyper-proliferative lesions in cervical, anogenital and oropharyngeal tissues. Our previous studies revealed that HPV16 E7 alters actin cytoskeleton mainly by binding to and inhibiting Gelsolin. We suggest that the physical interaction E7/Gelsolin in HPV positive tumor cell, and the resulting epithelial to mesenchymal transition process, induce HIPPO signaling cascade by promoting YAP inactivation and favoring HPV-induced cell transformation, cancer motility and aggressiveness. The results of this study provide new insights into the oncogenic transformation mechanisms elicited by HPV in the infected cells and may suggest a repertoire of targets for therapeutic purposes.

**Abstract:**

Human papillomavirus 16 (HPV16) exhibits a strong oncogenic potential mainly in cervical, anogenital and oropharyngeal cancers. The E6 and E7 viral oncoproteins, acting via specific interactions with host cellular targets, are required for cell transformation and maintenance of the transformed phenotype as well. We previously demonstrated that HPV16E7 interacts with the actin-binding protein gelsolin, involved in cytoskeletal F-actin dynamics. Herein, we provide evidence that the E7/gelsolin interaction promotes the cytoskeleton rearrangement leading to epithelial-mesenchymal transition-linked morphological and transcriptional changes. E7-mediated cytoskeletal actin remodeling induces the HIPPO pathway by promoting the cytoplasmic retention of inactive P-YAP. These results suggest that YAP could play a role in the “de-differentiation” process underlying the acquisition of a more aggressive phenotype in HPV16-transformed cells. A deeper comprehension of the multifaceted mechanisms elicited by the HPV infection is vital for providing novel strategies to block the biological and clinical features of virus-related cancers.

## 1. Introduction

Human papillomaviruses (HPVs) are small, non-enveloped, double-stranded circular DNA viruses, which cause hyperproliferative lesions in cutaneous and mucosal epithelial tissues [1,2]. Approximately 70% of all HPV-positive squamous cervical carcinomas are due to high-risk HPV16 or HPV18 infection [3], with persistent expression of the HPV-encoded E6 and E7 oncoproteins, which are essential for the establishment and maintenance of the transformed cellular phenotype. Both E6 and E7 multifunctional viral proteins lack enzymatic activity and are able to perturb several critical host cell pathways via specific protein–protein interactions with a plethora of cellular factors, thus promoting cancer onset and progression [4,5].

In particular, E6 and E7 stimulate epithelial-mesenchymal transition (EMT), a crucial step for carcinogenic progression [6,7]. The dynamic remodeling of actin filaments is fundamental for many processes involved in cancer progression and metastatization, as cell motility, proliferation, apico-basal polarity, invasive potential, intracellular transport and adhesion [8]. Moreover, cancer cells undergoing EMT reorganize the architecture of the actin cytoskeleton and dissolve adherent and tight junctions, thus losing cell−cell contacts and apico-basal polarity [9]. HPV16E7 plays a key role in regulating cell migration and invasion, thus promoting progression and metastasis of HPV-positive cancers [6,10]. In a previous study, we showed that HPV16E7 alters the actin cytoskeleton by binding to and inhibiting the calcium-regulated cellular factor, gelsolin (GSN), involved in severing, capping and nucleation of actin cytoskeletal filaments [11]. This protein−protein interaction promotes reorganization of the actin cytoskeleton leading to a more aggressive cancer phenotype [7].

Actin cytoskeleton functioning plays a central role in both developmental and cancer-related EMT in epithelial and mesenchymal cells [12]. In particular, the small guanosine triphosphatase (GTPase) family members, including RhoA, Rac1 and Cdc42 RhoGTPases, actively control actin−myosin dynamics [13].

Actin fiber dynamics and resulting apical cortical tension exert a strong impact on the tumor suppressor HIPPO pathway [14,15,16] by affecting nucleo-cytoplasmic shuttling of its downstream effectors, i.e. Yes-associated protein (YAP), the YAP paralog with PDZ-binding motif (TAZ), and the transcriptional activity of TEAD (TEA/ATTS domain) complexes [17,18]. Mechanisms regulating YAP activity through mechano-transduction involve modulation of intracellular forces by cytoskeletal fibers between focal adhesions and cell nucleus [19]. 

Moreover, mounting evidence supports a dual role of YAP as either oncogenic or oncosuppressor protein, depending on its binding partners and subcellular localization [20,21].

Starting from this background, in this study we identified and characterized some cellular mechanisms induced by HPV16E7 expression responsible for the actin cytoskeletal rearrangement and associated with aggressive tumor phenotype. We provide evidence that the E7–GSN physical interaction and the resulting EMT process induce the HIPPO kinase cascade by promoting YAP inactivation through cytoplasmic retention of its phosphorylated form, thus favoring HPV-induced cell transformation and cancer progression.

## 2. Materials and Methods

### 2.1. Cell Line and Treatments

C33A (HPV-) human cervical cancer cell line (American Type Culture Collection ATCC; Rockville, MD, USA) was cultured in DMEM (Invitrogen Corporation, Carlsbad, CA, USA) supplemented with 10% FBS (Euroclone, Milan, Italy). In RhoGTPases inhibition assays, C33A cells were treated with 15 µM Y27632 (Sigma-Aldrich, St Louis, MO, USA) for 40 min, while in actin inhibition assays cells were treated with 1 mM Cytochalasin D (CytoD, Sigma-Aldrich, St Louis, MO, USA) for 4 h. Cells treated with vehicle alone (0.1% DMSO) were used as control. 

### 2.2. Plasmids and Cell Transfection

pAmCyan-HPV16E7wt and HPV16E7 deletion mutant constructs (pAmCyan-HPV16E7∆62-66 and pAmCyan-HPV16E7∆71–75) were generated according to Mileo et al. [11]. All plasmids were transfected using Lipofectamine2000 (Invitrogen, Carlsbad, CA, USA) according to manufacturer’s instruction. Control cells (Mock) were transfected with pAmCyan1-C1 (Clontech Laboratories Inc., Mountain View, CA, USA) empty vector. For GSN silencing assay, FlexiTube siRNA mix for GSN (#SI02664039 and #SI02664046) and negative control siRNA (#1022076) were purchased from Qiagen (Qiagen Corporation, Gaithersburg, MA, USA). C33A cells were co-transfected with DNA plasmids and siRNAs according to Qiagen instructions. The transfected cells were then cultured for 48 h before processing. 

### 2.3. RNA Extraction and Quantitative RT-PCR 

Total RNA was extracted from C33A cells using the MasterPure RNA Purification Kit (Epicentre Biotechnologies, Madison, WI, USA). RNA was reverse-transcribed into cDNA using the High-Capacity cDNA Reverse Transcription Kit (Applied Biosystems Inc., Foster City, CA, USA) and subject to StepOne Real-Time PCR (Applied Biosystems Inc., Foster City, CA, USA) with PowerUp SYBR Green Master Mix (Applied Biosystems Inc., Foster City, CA, USA). Primers for Cyr61, CTGF, Zeb1, Snail and GAPDH were designed as specified below: 

Cyr61 forward 5′-GAGTGGGTCTGTGACGAGGAT-3′

Cyr61 reverse 5′-GGTTGTATAGGATGCGAGGCT-3′

CTGF forward 5′-AATGCTGCGAGGAGTGGGT-3′

CTGF reverse 5′-CGGCTCTAATCATAGTTGGGTCT-3′

Zeb1 forward 5′-GCAGTCCAAGAACCACCCTT-3′

Zeb1 reverse 5′-GGGCGGTGTAGAATCAGAGT-3′

Snail forward 5′-ACTATGCCGCGCTCTTTCC-3′

Snail reverse 5′-GTCGTAGGGCTGCTGGAAG-3′

GAPDH forward 5′-TCCCTGAGCTGAACGGGAAG-3′

GAPDH reverse 5′-GGAGGAGTGGGTGTCGCTGT-3′

PCR conditions were 50 °C for 2 min, 95 °C for 2 min, followed by 40 cycles of 95 °C /15 s, annealing at 56 °C/30 s and 72 °C/30 s. All reactions were performed in triplicate. Data were normalized to GAPDH and the fold change in gene expression relative to normal was calculated using the comparative *Ct* method [22].

### 2.4. Cell Invasion Assay

Transwell cell invasion assay was performed using Matrigel-coated inserts (8.0 μm pore size) following manufacturer’s instruction (Becton Dickinson, Franklin Lakes, NJ, USA). Each assay was carried out at least three-times in triplicate for each experimental condition. 1 × 105 cells/mL were placed in the upper compartment of the chamber of 24-well transwell culture inserts. Medium with 20% FCS was used as chemoattractant in the lower compartment of the chamber. The plates were incubated at 37 °C in a 5% CO_2_ atmosphere for 48 h. At the end of the incubation, the cells on the upper surface of the filter were completely removed by wiping with a cotton swab. pAmCyan transfected cells in the lower surface of the membrane were quantitatively evaluated with a fluorescence microscope (20× objective). 

### 2.5. RhoGTPases Activity

Activation of RhoGTPases was determined with the Rho Activation Assay Combo Kit (Cell Biolabs, San Diego, CA, USA; #STA-405,). After lysis, western blotting according to the manufacturer protocol was performed to assess RhoGTPases activation.

### 2.6. Immunoblot Analysis

The whole-cell extract was obtained in RIPA buffer in the presence of standard protease and phosphatase inhibitors. The protein content was determined with a protein assay reagent (Bio-Rad Laboratories Inc., Hercules, CA, USA), using bovine serum albumin as a standard. Equal protein content of total cell lysates was resolved on polyacrylamide gel (Bolt 4–12% Bis-Tris Plus Invitrogen, Carlsbad, CA, USA), electro-transferred to PVDF membranes (iBlot Invitrogen, Carlsbad, CA, USA) and incubated with specific primary antibodies: anti-YAP (Santa Cruz Biotechnology, Inc., Dallas, TX, USA; sc #101199; dil.1:1000); anti-p-YAP (Cell Signaling Technology, Inc., Beverly, MA, USA; #13008; dil.1:1000); anti-β Actin (MP Biomedicals, Santa Ana, CA, USA; # 69100; dil.1:10000); anti-Rac1, anti-RhoA and anti-Cdc42 (all Cell Biolabs, San Diego, CA, USA; STA-405; dil.1:500); anti-Zeb1 (Abcam, Cambridge, MA, USA; #180905; dil.1:2000); anti-Snail1 (Santa Cruz Biotechnology, Inc., Dallas, TX, USA; sc-271977; dil.1:1000); anti-GSN (Sigma-Aldrich, St Louis, MO, USA; G-4896, dil.1:1000). Membranes were developed using ECL detection reagents (GE Healthcare Life Sciences, Uppsala, Sweden) on a UVITEC imaging system (UVITEC, Cambridge, UK) or ChemiDocMP system (Bio-Rad Laboratories Inc.). Quantitative analysis of western blots was performed using ImageJ (NIH) software [23].

### 2.7. Flow Cytometry

#### 2.7.1. Quantitative Evaluation of Proteins

Cells were fixed with 4% paraformaldehyde (Carlo Erba, Milan, Italy), permeabilized by 0.5% Triton X-100 (Sigma-Aldrich, St Louis, MO, USA) and incubated for 1 h at 4 °C with the following antibodies, at a final concentration of 0.1 mg/mL: anti-AMOT1 (Santa Cruz Biotechnology, Inc. Dallas, TX, USA; sc-166924), anti-YAP (Santa Cruz Biotechnology, Inc., Dallas, TX, USA) or anti-p-YAP (Cell Signaling Technology). For F-actin detection, cells were stained with Biotin-XX Phalloidin (Thermo Fisher Scientific, Waltham, MA, USA; #B7474). After washings, cells were incubated for 30 min at 37 °C with a secondary antibody conjugated with Cy5 (Abcam) or Streptavidin-Cy5 (Thermo Fisher Scientific, Waltham, MA, USA). Cell samples were washed twice in PBS and immediately acquired by a cytometer.

For flow cytometry studies, samples were acquired with a FACScalibur cytometer (BD Biosciences Inc., San Diego, CA, USA) equipped with a 488 nm argon laser and with a 635 nm red diode laser. At least 20,000 events were acquired, recorded and analyzed using CellQuest software (BD Biosciences, San Diego, CA, USA). The expression level of the analyzed proteins by flow cytometry was reported as median fluorescence.

#### 2.7.2. Fluorescence Resonance Energy Transfer (FRET)

We applied quantitative FRET analysis by flow cytometry to C33A cells transiently transfected with HPV16E7wt or HPV16E7-mutant constructs. FRET analysis was restricted to cells positive to FL-1 (corresponding to pAmCyan1-C1 fluorescence emission). Cells were fixed, permeabilized and labeled as previously reported [24]. For FRET analyses, we used: anti-AMOT1 (Santa Cruz Biotechnology, Dallas, TX, USA), anti-p-YAP, anti-PTPN14 (Invitrogen, Carlsbad, CA, USA; MA5-31871), HPVE16-E7 polyclonal antibody (Bioss Antibodies, Woburn, MA, USA; bs-10446R) and Biotin-XX Phalloidin (Thermo Fisher Scientific, Waltham, MA, USA), PE-labeled anti-mouse (Sigma-Aldrich, St Louis, MO, USA), and Cy5-labeled anti-rabbit (Thermo Fisher Scientific, Waltham, MA, USA). AMOT1 protein was detected in the FL2 channel (PE, donor), p-YAP and F-actin in FL4 (Cy5, acceptor), and FRET in FL3 channel. 

Quantification of protein−protein interaction was obtained by calculating FRET efficiency (FE) by using the following Riemann algorithm [25]:

FE = (FL3DA − FL2DA/a − FL4DA/b)/FL3DA in which A is the acceptor and D the donor and where a = FL2D/FL3D and b = FL4A/FL3A.

### 2.8. Fluorescence Microscopy

Cells were fixed with 4% paraformaldehyde, permeabilized by 0.5% (*v*/*v*) Triton X-100 [24] and incubated for 1 h at 4 °C with primary antibodies. The following primary and secondary antibodies were used: anti-AMOT1, anti-YAP, anti-p-YAP, AlexaFluor 488-conjugated anti-rabbit (Invitrogen, Carlsbad, CA, USA; #A11034), AlexaFluor 488-conjugated anti-mouse IgG (Invitrogen, Carlsbad, CA, USA; #A11001) and AlexaFluor 594-conjugated anti-mouse IgG (Invitrogen, Carlsbad, CA, USA; #A11005). For F-actin detection, cells were stained with TRITC-Phalloidin (Sigma-Aldrich, St Louis, MO, USA; #P1951) for 30 min at room temperature. After washing, all samples were counterstained with Hoechst 33258 (Sigma-Aldrich, St Louis, MO, USA; #861405) and then mounted in fluorescence mounting medium (Dako, Glostrup, Denmark; #S3023). Images were acquired by intensified video microscopy (IVM) with an Olympus fluorescence microscope (Olympus Corporation of the Americas, Center Valley, PA, USA), with a Zeiss charge-coupled device camera (Carl Zeiss, Oberkochen, Germany).

### 2.9. Data Analyses and Statistics

For flow cytometry studies, at least 20,000 events were acquired. Data were recorded and statistically analyzed by a Macintosh computer using CellQuest software (BD Biosciences, San Diego, CA, USA). The expression level of the analyzed proteins by flow cytometry was expressed as median fluorescence. Collected data analysis was carried out by ANOVA 2-way testing for repeated samples, using GraphPad Prism 5 software (GraphPad, San Diego, CA, USA). All data were verified in at least three independent experiments and are reported as means ± standard deviation (SD). *p* < 0.05 was considered to be statistically significant (* *p* < 0.05, ** *p* < 0.01, *** *p* < 0.001).

## 3. Results

### 3.1. HPV16E7 Expression Promotes Actin Cytoskeleton Remodeling, Cell Invasion and EMT in Human Cervical Cancer Cells 

**Figure 1:**
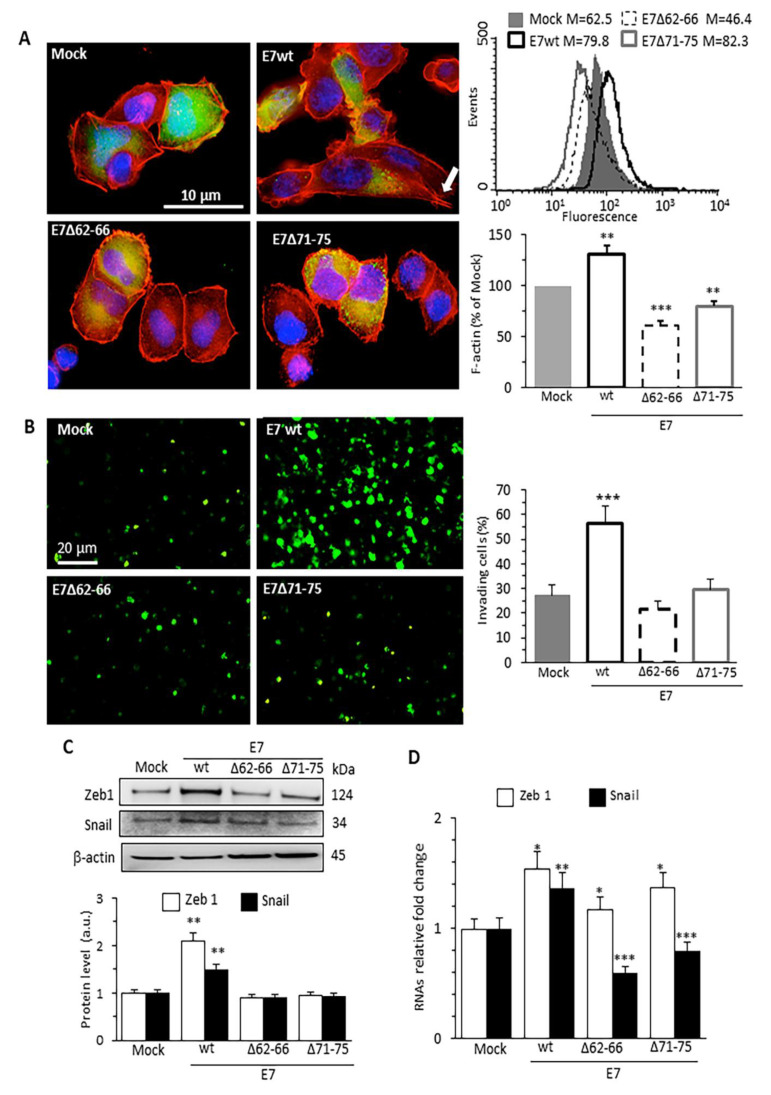
HPV16E7 expression promotes actin cytoskeleton remodeling, cell invasion ability and EMT in human cervical cancer cells. (**A**) IVM analysis after double-cell staining with TRITC-phalloidin (red) and Hoechst (blue) in C33A cells transfected (green) with pAmCyan empty vector (Mock), pAmCyan-HPV16E7 wild-type (E7wt) or deletion (E7mut) mutant constructs pAmCyan-HPV16E7Δ62–66 (E7Δ62–66) or pAmCyan-HPV17E7Δ71–75 (E7Δ71–75). E7wt-expressing cells show different morphological features compared to both Mock- and E7 mutant transfected cells. Arrow indicates protrusive and invasive structures (left). Flow cytometry evaluation of the intracellular amount of F-actin restricted to pAmCyan-positive cells. Histograms obtained in a representative experiment are shown. Bar graph shows the mean ± SD of the median fluorescence intensity obtained in four different experiments. ** *p* < 0.01 and *** *p* < 0.001 vs. Mock transfected cells (right). (**B**) Representative images of transwell invasion assay of pAmCyan C33A transfected cells (left). Fluorescence emission of C33A migrating through Matrigel are quantified by IVM analysis and expressed as percentage (right). (**C**) Western blot analysis of the expression of the EMT markers Zeb1 and Snail. β−actin determination was used as loading controls. Bar graph (bottom) shows relative densitometry quantitation of each protein normalized to β-actin obtained in three independent experiments and reported as mean ±SD. ** *p* < 0.01 vs. Mock transfected cells. Uncropped western blot figure available in Supplementary Figure S5. (**D**) Bar graph showing the evaluation of mRNA levels of Zeb1 and Snail performed by qRT-PCR assay. Data are reported as mean ±SD of RNAs relative fold change vs Mock-transfected cells obtained in three independent experiments. * *p* < 0.05, ** *p* < 0.01 and *** *p* < 0.001 vs. Mock transfected cells.

In order to assess the impact of HPV16E7 expression on cytoskeleton actin polymerization status, we used the C33A, HPV-null, human cervical carcinoma cell line to express HPV16E7 wild-type (wt) or its deletion mutants HPV16E7Δ62–66 (E7Δ62–66) and HPV16E7Δ71–75 (E7Δ71–75), both unable to bind to the GSN molecule [7,11] (henceforth called E7 mutants). Specifically, the C33A cell line was chosen as experimental model to evaluate the HPV16E7 expression effects, in the absence of other viral proteins, but in a cellular context of cervical cancer. Ectopic E7wt expression in C33A increased exclusively the amount of filamentous actin (F-actin) fraction. By contrast, E7 mutants determined a fairly consistent reduction of cellular F-actin amount compared with either Mock- or E7wt-transfected cells. Fluorescence images of TRITC-Phalloidin/Hoechst double staining of C33A cells showed cytoskeletal remodeling due to E7wt expression (Figure 1A). E7wt-expressing cells showed morphological features and increased amount of F-actin resulting in gaining protrusive and invasive structures, possibly associated with cell elongation and activation of directional motion dynamics. Accordingly, flow cytometry analysis, restricted to pAmCyan-positive (i.e., efficiently transfected) cells, revealed an increased amount of F-actin in E7wt compared with Mock-transfected cells, while cells transfected with E7 mutants showed a significant F-actin reduction (Figure 1A bar graph on the right). This E7-mediated cytoskeletal actin remodeling was coupled with a significant increase in cellular invading properties, measured as the ability of these cells to cross the Matrigel layer, which appeared definitely increased in E7wt-expressing cells, when compared with either Mock-transfected or E7 mutant-expressing cells (Figure 1B).

Western blot analyses (Figure 1C) and RT-PCR (Figure 1D) of two EMT-related transcription factors, Zeb1 and Snail, which are powerful regulators of the epithelial genes involved in cell motility and intercellular adhesion, indicated their significant increase in E7wt-transfected cells and a less evident increase in E7 mutant-transfected cells. Thus, E7wt expression appears to promote both EMT and cell invasion through a cytoskeletal actin remodeling mediated by the interaction of the oncoproteins with GSN. 

### 3.2. HPV16E7 Expression Induces Activation of Rac1 and Cdc42 RhoGTPases 

**Figure 2:**
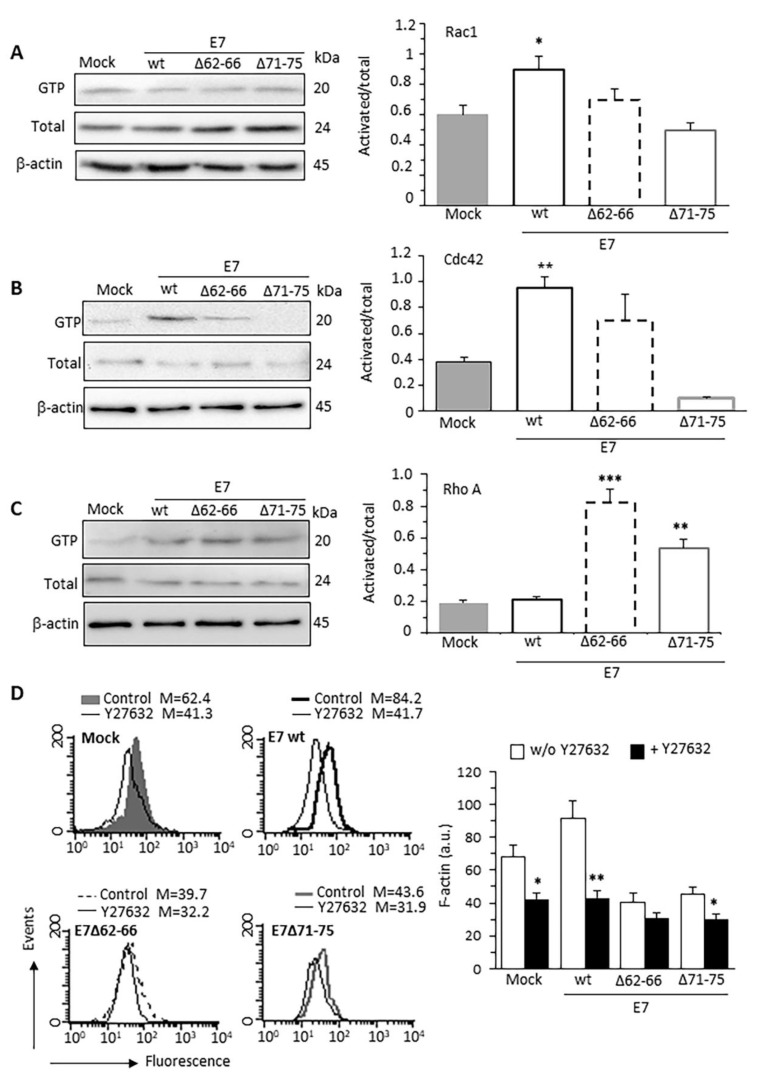
HPV16E7 expression induces activation of Rac1 and Cdc42 GTPases. Activation state of RhoGTPases measured by pull-down assays using PBD domain of PAK for Rac1 (**A**) or Cdc42 (**B**), and the RBD domain of Rhotekin for RhoA (**C**), followed by immunoblotting with the respective antibodies. In addition, Rac1, Cdc42, or RhoA from total lysates were quantified (left panels). In the right panels, bar graph shows the active forms of Rac1, Cdc42, and RhoA GTP (GTP-bound levels/total levels) normalized to β-actin. The mean ± SD of the results obtained in three independent experiments is shown. * *p* < 0.05, ** *p* < 0.1 and *** *p* < 0.001 vs. Mock transfected cells. (**D**) Flow cytometry evaluation of the intracellular amount of F-actin, restricted to pAmCyan-positive cells, before or after treatment with the RhoGTPase inhibitor Y27632 (15 µM for 40 min). Bar graph (right) reports results as mean ± SD of the median fluorescence intensity obtained in three different experiments. * *p* < 0.05 and ** *p* < 0.01, vs the same sample treated with Y27632. Uncropped western blot images for Figure 2A–C available in Supplementary Figure S6.

We checked the activation status of Rac1, RhoA and Cdc42, which are all factors involved in EMT mainly by perturbing actin rearrangement and intercellular adhesion structures [26,27]. Increased Rac1 activation was evident in E7wt-expressing cells, when compared with Mock-transfected cells (Figure 2A). Conversely, E7 mutant-expressing cells displayed non-significant variations in Rac1 activity. Cdc42 displayed a similar trend, being activated in E7wt cells (Figure 2B). RhoA activity was not influenced by E7wt but appeared particularly stimulated in cells transfected with both E7 mutants, in line with the inhibitory role of RhoA on Rac1 activity (Figure 2C) [28,29].

We, therefore, restricted the analysis of F-actin content to fluorescence-positive cells treated with the RhoGTPases inhibitor Y27632 (15 μM for 40 min) or left it untreated. Y27632 was able to reduce F-actin content in E7wt-expressing cells and, to a minor extent, in Mock-transfected cells (Figure 2D). By contrast, the inhibitor resulted ineffective in cells transfected with E7 mutants, characterized by a reduced basal level of F-actin. These data show the functional involvement of RhoGTPases in the F-actin alterations mediated by the E7–GSN interaction.

### 3.3. HPV16E7 Expression Promotes Phosphorylation of YAP

**Figure 3:**
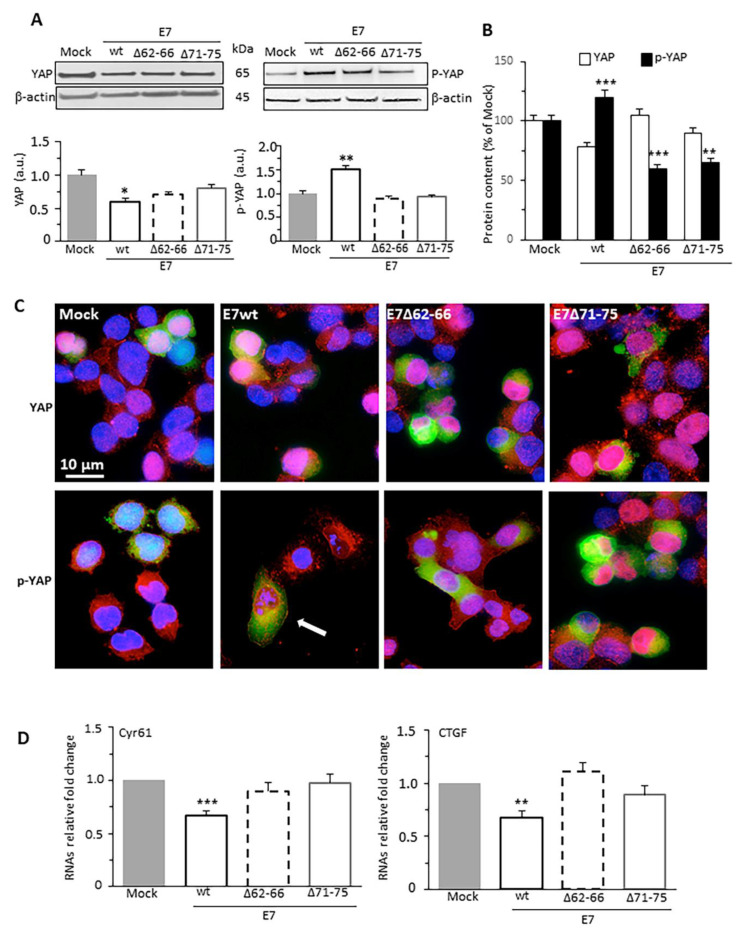
HPV16E7 promotes phosphorylation of YAP. (**A**) Western blot analysis of the expression level of YAP and p-YAP in Mock-transfected cells, and in cells transfected with E7wt or deletion mutant constructs E7Δ62–66 or E7Δ71–75. β−actin determination was used as loading controls. Bar graph (bottom) shows relative densitometry quantitation of each protein normalized to β-actin obtained in three independent experiments. * *p* < 0.05 and ** *p* < 0.01, vs. Mock transfected cells. (**B**) Bar graph showing flow cytometry evaluation of YAP and p-YAP amount restricted to pAmCyan-positive cells. Results are the means ± SD of the median fluorescence intensity obtained in four different experiments. ** *p* < 0.01, and *** *p* < 0.001, vs. Mock transfected cells. (**C**) IVM analysis after double cell staining with anti-YAP (upper line) or anti-p-YAP antibody (bottom line) (both in red) and Hoechst (blue) of cells transfected (green) with pAmCyan empty vector (Mock), E7wt or deletion mutant constructs E7Δ62–66, E7Δ71–75. Interestingly, p-YAP is found exclusively in the cytoplasm only in E7wt cells (yellow fluorescence, see arrow). (**D**) Bar graphs showing the evaluation of mRNA levels of Cyr61 (left) and CTGF (right), two downstream YAP target genes, performed by real-time qRT-PCR assay. Data are reported as mean ± SD of RNAs relative fold change vs Mock-transfected cells, obtained in three independent experiments. ** *p* < 0.01 and *** *p* < 0.001 vs. Mock transfected cells. Uncropped western blot images for Figure 3A available in Supplementary Figure S7.

Being actin microfilament system a crucial mediator in the regulation of HIPPO signaling [30,31,32], we checked whether F-actin rearrangement mediated by HPV16E7 expression implies involvement of YAP, the main downstream transcriptional co-activator of the HIPPO tumor suppressor pathway. Since YAP activity is mainly regulated by serine127 phosphorylation [33,34], we have assessed YAP/p-YAP expression ratio in C33A cells expressing HPV16E7 isoforms. Western blot analysis indicated that p-YAP amount was higher in E7wt-expressing cells compared with controls. By contrast, a decrease in p-YAP was observed in cells transfected with E7 mutants (Figure 3A). It has been widely demonstrated that YAP inhibition is also promoted by high cell density [33]. In order to avoid this bias and to confirm the crucial role of E7–GSN binding on YAP activity, we performed all assays on cells cultured at low and constant density (30–40% confluence). In these experimental conditions, in the control cells, HIPPO signaling is constitutively inactive, while YAP moves to the nucleus to function as a transcriptional coactivator. Our western blot data were consistent with those obtained via flow cytometry when the analysis was restricted to the cells effectively transfected (Figure 3B). Notably, E7wt expression induced YAP phosphorylation, thus promoting its cytoplasmic retention and inactivation. Furthermore, E7wt-expressing cells displayed a reduced YAP content, in agreement with the parallel increase of YAP phosphorylation level, suggesting that the cytoplasmic YAP fate may be the ubiquitin-mediated proteasomal degradation [17]. Since the HIPPO pathway negatively regulates YAP activity mainly by promoting its cytoplasmic retention, we have investigated its cellular localization by immunofluorescence staining (Figure 3C). We observed an increase of p-YAP cytoplasmic localization in E7wt-expressing cells compared with cells expressing E7 mutants (Figures S1 and S2). The YAP/p-YAP molecular ratio, which represents the nuclear activity of the HIPPO-regulated transcriptional factor, was consistently higher in C33A cells expressing E7 mutants than those expressing E7wt. To evaluate the effect of HPV16E7 expression on YAP transcriptional activity, using qRT-PCR we checked the mRNA levels of two downstream YAP target genes, the connective tissue growth factor (CTGF) and cysteine-rich angiogenic protein 61 (Cyr61). In line with previous published data [35], both Cyr61 and CTGF mRNA levels were downregulated in E7wt-expressing cells, in accordance with the observed increase in p-YAP amount (Figure 3D). Conversely, cells expressing E7 mutants displayed substantially unmodified levels of both factors. 

### 3.4. Specific Relevance of the E7–GSN Interaction 

**Figure 4:**
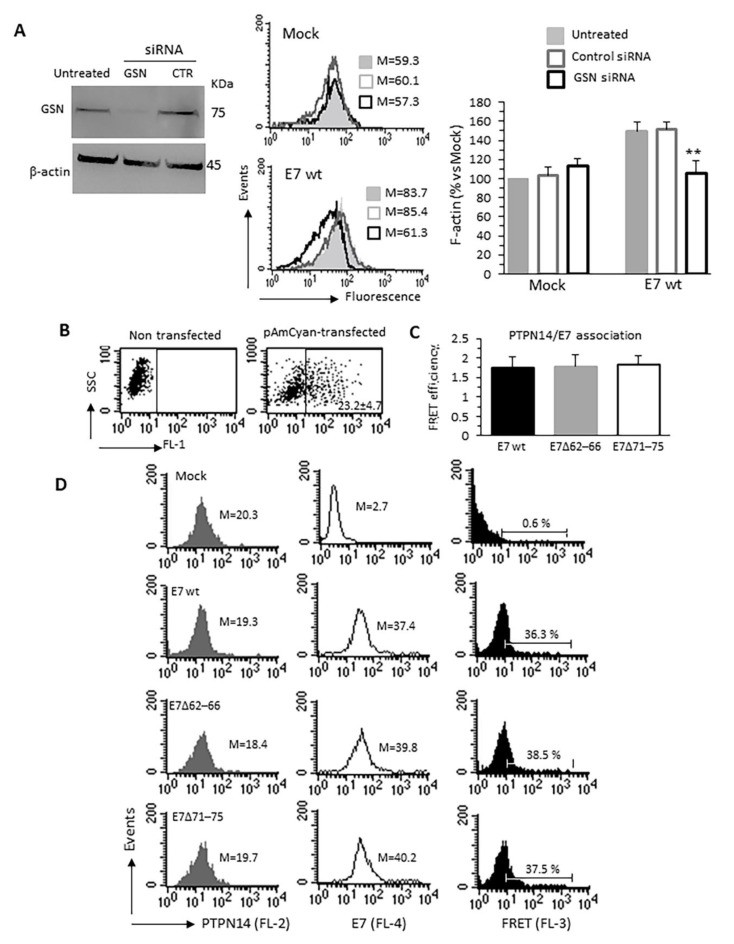
Specific relevance of E7-GSN interaction. (**A**) Representative western blot of endogenous gelsolin (GSN) knockdown showing the relative abundance of GSN in pAmCyan trasfected cells (Mock), in transfected cells treated with specific siRNAs for GSN silencing (siRNAGSN) or treated with scrambled siRNAs as a negative control (siRNAcontrol) (left). Flow cytometry evaluation of the intracellular amount of F-actin restricted to pAmCyan-positive cells transfected with siRNAGSN or scrambled siRNA as control. Histograms obtained in a representative experiment are shown. Bar graph (right) shows the mean ± SD of the median fluorescence intensity obtained in four different experiments. ** *p* < 0.01 and *** *p* < 0.001 vs. Mock transfected cells. (**B**–**D**) Quantitative evaluation of PTPN14/E7 molecular association by FRET technique. (**B**) Flow cytometry analysis was restricted to pAmCyan-positive cells (reported as percentage). Bar graph in (**C**) shows FRET efficiency calculated according to the Riemann’s algorithm. Data are reported as mean ± SD from three independent acquisitions. (**D**) Cytofluorimetric histograms obtained in one experiment representative of three. Numbers in the first and second columns indicate the median fluorescence intensity of the respective proteins, the percentage in the third column represent FL3 positive events. Uncropped western blot images for Figure 4A are available in Supplementary Figure S8.

To assess the contribution of the interaction between E7 and GSN in producing the results described above, we silenced GSN expression in C33A cells by transfecting them with a mixture of specific siRNAs. Figure 4A, highlights, by means of western blot, that these siRNAs, but not the control ones, substantially down-regulated GSN expression in pAmCyan-positive (efficiently transfected) cells and rendered ineffective E7wt in increasing the cellular F-actin content.

Furthermore, since PTPN14, an interactor of the HPV16E7 C-terminal region, has been characterized as a negative regulator of YAP [35], to rule out the involvement of a HPV16E7–PTPN14 interaction on YAP signaling in our experimental setting, we assayed the ability of E7wt and its two C-terminal mutants to bind PTPN14. Fluorescence Resonance Energy Transfer (FRET) assay showed that either E7wt or E7 mutants bound PTPN14 with the same efficacy (Figure 4B–D), thus indicating that the E7-mediated YAP phosphorylation was related to the interaction of E7 with GSN rather than with PTPN14. 

### 3.5. HPV16E7-Induced Cytoskeleton Alterations Interfere With p-YAP/AMOT1 Complex

**Figure 5:**
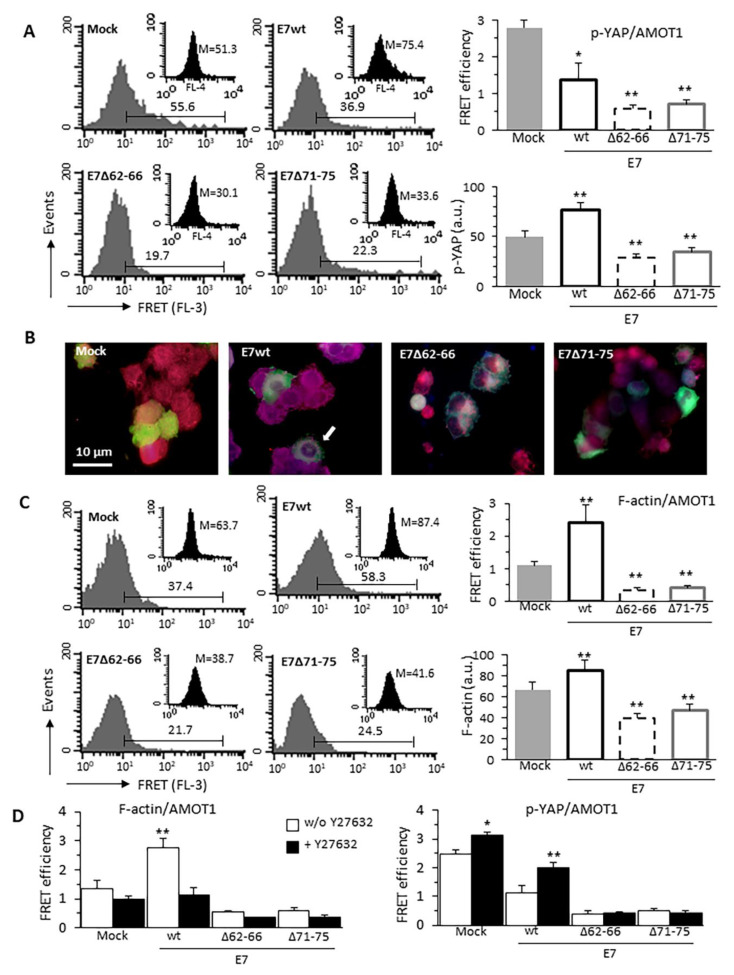
E7-induced F-actin alterations interfere with p-YAP/AMOT1 interactions. Quantitative evaluation of (**A**) p-YAP/AMOT1 and (**C**) F-actin/AMOT1 molecular association by FRET technique, as revealed by flow cytometry analysis restricted to pAmCyan-positive cells. Numbers indicate the percentage of FL3-positive events, obtained in one experiment representative of three. Bar graphs on right show FRET efficiency calculated according to the Riemann’s algorithm. Data are reported as mean ± SD from three independent experiments. Inset: representative flow cytometry histograms of p-YAP expression (**A**) or F-actin amount (**C**) in the corresponding sample. Numbers indicate the median fluorescence intensity. Bar graphs on the bottom right represent the mean ± SD of the median fluorescence intensity values obtained in three different experiments. * *p* < 0.05 and ** *p* < 0.01 vs. Mock transfected cells. (**B**) IVM analysis after double cell staining with anti-p-YAP (blue) and anti-AMOT antibody (red) of cells transfected (green) with pAmCyan empty vector (Mock), E7 wild-type (E7wt) or deletion mutant constructs E7Δ62–66, E7Δ71–75. To note that p-YAP/AMOT complexes appeared markedly distributed in the perinuclear area of E7wt cells while were reduced and scattered in the cytoplasm of mutant cells (see arrow); (**D**) Bar graphs showing F-actin/AMOT1 (left) and p-YAP/AMOT1 (right) molecular association observed in different samples treated or not with the RhoGTPase inhibitor Y27632 and quantified as FRET efficiency. Analysis was restricted to pAmCyan-positive cells. Data are reported as mean ± SD from three independent experiments.

YAP directly binds the Amot p130 isoform (AMOT1) [36,37,38]. The N-terminal domain of AMOT1 harbors three proline-rich PPxY (PY) motifs that are involved in both YAP and F-actin binding. Thus, we investigated the effect of HPV16E7 expression on p-YAP/AMOT1 and F-actin/AMOT1 physical interactions. FRET assay, performed on FL-1-positive cells only, showed that the molecular association p-YAP/AMOT1 was reduced in E7wt cells rather than in Mock-transfected cells and, although to a lesser extent, in E7 mutants expressing cells (Figure 5A). Immunofluorescence images (Figure 5B) showed a marked co-localization of p-YAP and AMOT1 in a cytoplasmic area around the nucleus in cells expressing E7wt. Conversely, in Mock-transfected cells, the co-localization of fluorescence signals appeared scattered throughout the cytoplasm, while in cells expressing both E7 mutants it appeared polarized in a cytoplasmic area close to the nucleus (Figure S3). The F-actin/AMOT1 complexes, increased in E7wt cells, were reduced in E7 mutant-expressing cells (Figure 5C). AMOT1 was expressed at relatively constant levels in all samples (Table 1), thus suggesting that the increase in F-actin levels, due to the expression of E7wt able to complex GSN, favored F-actin/AMOT1 association. Consequently, in cells expressing E7 mutants, a reduction of the molecular interaction between F-actin and AMOT1 was evident, also compared with Mock-transfected cells. 

To establish the role of microfilament system polymerization state in the formation of F-actin/AMOT1 complexes, transfected C33A cells were treated with the RhoGTPase inhibitor Y27632. At FRET analysis, inhibition of RhoGTPase-dependent actin polymerization induced a decrease of F-actin/AMOT1 interaction that, although observed to some extent in all samples, reached significance only in E7wt-expressing cells (Figure 5D). In agreement, we detected a Y27632-induced increase of the p-YAP/AMOT1 complex in Mock- and E7wt-transfected cells, but not in those expressing E7 mutants (Figure 5C). These results suggest that E7–GSN interaction could regulate YAP also by modulating RhoGTPase activity. 

### 3.6. Effect of Cytochalasin D on YAP Activity and EMT

**Figure 6:**
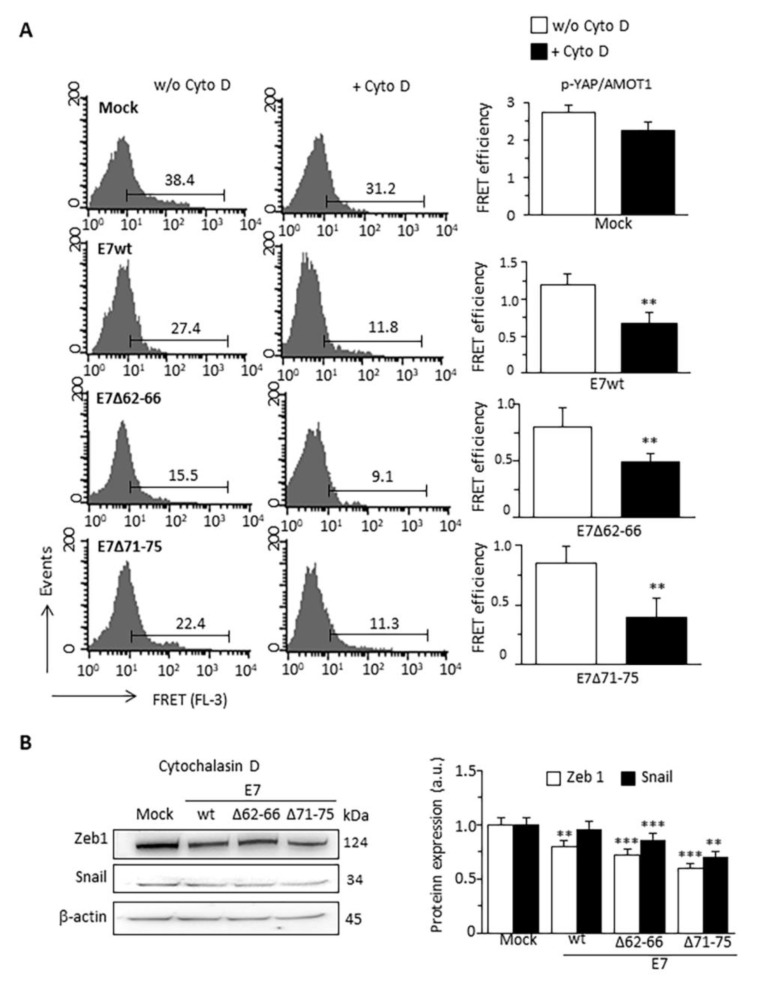
Effect of Cytochalasin D on p-YAP/AMOT1 interaction and expression of EMT markers. (**A**) Quantitative evaluation of p-YAP/AMOT1 molecular association by FRET technique, as revealed by flow cytometry analysis restricted to pAmCyan-positive cells treated or not with CytoD (1 mM for 4 h). Numbers indicate the percentage of FL3 positive events obtained in one experiment representative of three. Bar graphs on the right show FRET efficiency calculated according to the Riemann’s algorithm. Data are reported as mean ± SD from three independent experiments. ** *p* < 0.01 vs. Mock transfected cells. (**B**) Western blot analysis of the expression of the EMT markers Zeb1 and Snail in C33A expressing E7wt or E7 mutated treated with CytoD. β-actin determination was used as loading controls. Bar graph (right) shows relative densitometry quantitation of each protein normalized to β-actin obtained in three independent experiments and reported as mean ± SD. ** *p* < 0.01, *** *p* < 0.001 vs the same sample treated with CytoD. Uncropped western blot images for Figure 6B are available in Supplementary Figure S9.

Since actin cytoskeleton architecture regulates the YAP activity [28,30], we tested the potential direct role of F-actin alterations on YAP activity by treating cells with cytochalasin D (CytoD), an actin polymerization inhibitor that disrupts the actin cytoskeleton and perturbs intracellular tensions. Cytofluorimetric analysis, performed on efficiently transfected cells only, showed that the increase of p-YAP determined by E7wt expression was counteracted by CytoD, which, by contrast, was unable to prevent p-YAP decrease induced by the expression of both mutant E7 isoforms (Table 1). In contrast, CytoD did not affect the expression of AMOT1. In addition, a significant reduction of the p-YAP–AMOT1 interaction level was observed in all samples after treatment with CytoD (Figure 6A). CytoD also prevented Zeb1 and Snail upregulation in E7wt-expressing cells (Figure 6B compared with Figure 1D; Figure S4). 

## 4. Discussion

The complex mechanism through which cells translate mechanical and physical stimuli into biological responses, called mechano-transduction, is relevant for cellular adaptation to dynamic changes in the microenvironment [39]. Indeed, nuclear-cytoskeletal coupling is crucial for the transmission of extra- and intra-cellular tensions to the nucleus and, consequently, for the biological response mediated by the expression of mechano-sensitive genes. However, the molecular mechanisms underlying these processes are largely unknown and scant information exists on the impact of onco-viral proteins on mechano-transduction processes and their effect on the acquisition of invasive and metastatic properties. Recently, the role of the adenovirus E1A oncoprotein in perturbing cell morphology and contributing to the acquisition of de-differentiated cell phenotype via its interference with the HIPPO pathway was reported [40]. E1A is an oncoprotein closely correlated with HPVE7 and exerts overlapping functional roles in the infected cells in order to favor cell transformation and maintenance of the malignant phenotype [41]. Interestingly, several comparative studies of the oncoproteins encoded by HPV, Adenovirus and Polyomavirus reveal similarities among their functions and cellular targets [42,43]. Furthermore, the repression of cell differentiation is a well-described oncogenic strategy mediated by E7 from high-risk HPV genotypes via a variety of RB-binding independent mechanisms [35,44]. Starting from these considerations and from our previous findings regarding the capability of HPV16E7 to hinder severing, capping and nucleation activity of the actin binding protein GSN, we explored the role of HPV16E7–GSN complex in actin microfilament rearrangement as a mechanical trigger for the acquisition of a more aggressive phenotype. Overall, quantitative and qualitative alterations in the F-actin cytoskeleton observed in HPV16E7-expressing cells appear closely related to EMT-linked morphological and transcriptional changes. Cancer cells undergoing EMT lose epithelial specialized membrane structures and properties (e.g., adherent/tight junctions and desmosomes), the apico-basal polarity and, consequently, acquire a mesenchymal, more aggressive, phenotype characterized by an elongated spindle-like cell shape, anchorage-independent cell growth, increased motility and invasiveness [45]. Several studies show the involvement of actin dynamics in the conversion of mechanical inputs into intracellular biochemical signals obtained by regulating the highly conserved HIPPO/YAP axis [19,46].

The role of YAP in cancerogenesis remains controversial. In fact, YAP activity promotes tumorigenicity [47,48] and, in other experimental models, apoptosis [49,50], suggesting that YAP integrates multiple morpho-functional signals driving to a transcriptional program that specifies cell fate [51]. The identification of the exact cellular context in which YAP promotes tumor growth rather than apoptosis is of great interest, but it remains unclear whether the mechanical cues promote YAP activity or whether the induction of YAP contributes to cancer cell cytoskeleton reorganization. 

Here, we investigated the relationship between the alteration of cytoskeletal architecture mediated by HPV16E7 expression and YAP activity. It should be highlighted that some reports indicate that YAP is inactivated through cytoplasmic retention by viral oncoproteins functionally related to HPVE7, thus contributing significantly to cell survival [52] and to the acquisition of a less differentiated phenotype in infected epithelial cells [40]. On the contrary, some studies report that YAP is hyperactivated and works as an oncogenic driver in HPV-related cancers [53,54,55] and that the HPV16 E6/E7 oncoproteins expression synergizes with YAP to promote cervical carcinogenesis [56].

Starting from this controversial scenario, we underline the role of the interaction between E7 and GSN in increasing YAP phosphorylation at serine127 and its consequent cytoplasmic retention. In fact, the basal cytoplasmic amount of p-YAP was not affected by the expression of the HPV16E7 mutants. Therefore, the E7–GSN interaction appears to play a pivotal role in reducing the transcriptional activity of YAP during EMT. 

The involvement of RhoGTPases in YAP activation has been extensively investigated, but its mechanism remains elusive [15,19,57]. The impact of cell morphology and mechanical stress on the HIPPO pathway may also be eliminated by blocking Rho activity, required to build-up the contractile network as cells react to a stiff matrix [14]. Indeed, RhoGTPases control the actin cytoskeleton functioning in epithelial and mesenchymal cells, with a major role in both developmental and cancer-related EMT [12]. Particularly, Rac1 and Cdc42 play a key role in the sub-membrane actin polymerization, invadopodia stability and cell-cell and cell-matrix relationships [58]. Moreover, RhoA and Rac1/Cdc42 activities, coordinated in regulating both membrane protrusions and cell matrix adhesions, have opposing effects on different modes of tumor cell motility [27,57].

From our data, it can be argued that E7 expression, via its interaction with GSN, may promote EMT transition and, therefore, mesenchymal motility mediated by the master regulators Cdc42 and Rac1. Conversely, in cells expressing E7 mutant isoforms, increased RhoA GTP could repress Rac1 activity, promoting an amoeboid-like movement of cancer cells. Data on the expression of EMT-related transcription factors, Zeb1 and Snail, involved in cell motility, seem to confirm the key role of the HPV16E7/GSN interaction in modulating EMT-linked mesenchymal motility. The Rac1/Cdc42 regulatory role on cytoskeletal actin appeared reduced in cells expressing E7 mutants. These findings strongly suggest the presence of an additional regulatory mechanism, GTPase-independent, in which GSN severing activity appears involved in the rearrangement of F-actin-mediated cytoskeleton in HPV16E7-expressing cells.

Angiomotin family members, mainly AMOT1, are regulators of transcriptional activity and subcellular localization of YAP through HIPPO-dependent [59] and independent [60] mechanisms. AMOT1, a junction-associated scaffold protein involved in endothelial polarization and migration, exhibits at its N-terminal region a F-actin binding site flanked by a YAP-binding motif. A competitive interaction of these two factors with AMOT1 regulates its ability to bind and sequester YAP in the cytoplasm [36,37] and activate YAP LATS kinase inhibitors of the HIPPO pathway [61]. Furthermore, AMOT1 inhibits the nuclear accumulation of YAP by binding it either to tight junctions or to actin filaments [36,37].

Moreover, increased actin polymerization, partly due to the impaired activity of GSN linked to its interaction with HPV16E7, promoted the association between F-actin and AMOT1. Although in cells expressing E7 mutants, the reduced availability of F-actin should favor the formation of p-YAP/AMOT complexes, this did not occur, possibly due to the rate-limiting p-YAP amount.

According to growing evidence highlighting the role of actin polymerization state in regulating YAP activity, FRET results indicated that the F-actin perturbation mediated by CytoD reduced the p-YAP–AMOT complexes formation, regardless the expression of HPV16E7, either wt or mutant. Interestingly, also in this condition of inhibited actin polymerization, cells showing HPV16E7–GSN interaction exhibited a higher propensity to inactivate YAP by increasing its phosphorylation and cytoplasmic sequestration. 

These data, in line with Fernandez et al. [62], suggest that F-actin level is per se determinant, although not conclusive, for YAP activation, while cytoskeletal remodeling mediated by E7–GSN interaction seemed to play a crucial role on HIPPO signature.

In order to define accurately the significance of the interplay between E7 and GSN in regulating YAP activity, we assayed the binding ability of E7wt and E7 mutants with PTPN14, another interactor able to modulate YAP activity [55,63]. Indeed, interaction with PTPN14 occurs at the level of the C-terminal zinc-finger domain of E7, the same region implicated in the binding with GSN [11]. In our experimental model, we confirmed the molecular interaction between PTPN14 and E7 described by White et al. [64], but the binding efficiency was not significantly different between E7wt and the C-terminal mutants. Therefore, the cytoplasmic retention of YAP due to the expression of E7 appears to be related with the E7–GSN complex rather than to the E7–PTPN14 interaction, unaffected by the E7 mutations described here.

The control of YAP function requires both Rac1 and Cdc42 activity, as well as the microfilament system tension due to actin polymerization promotion mediated by HPV16E7–GSN interaction, suggesting a crucial role of the HIPPO–YAP axis in the EMT-linked cytoskeleton remodeling.

**Figure 7:**
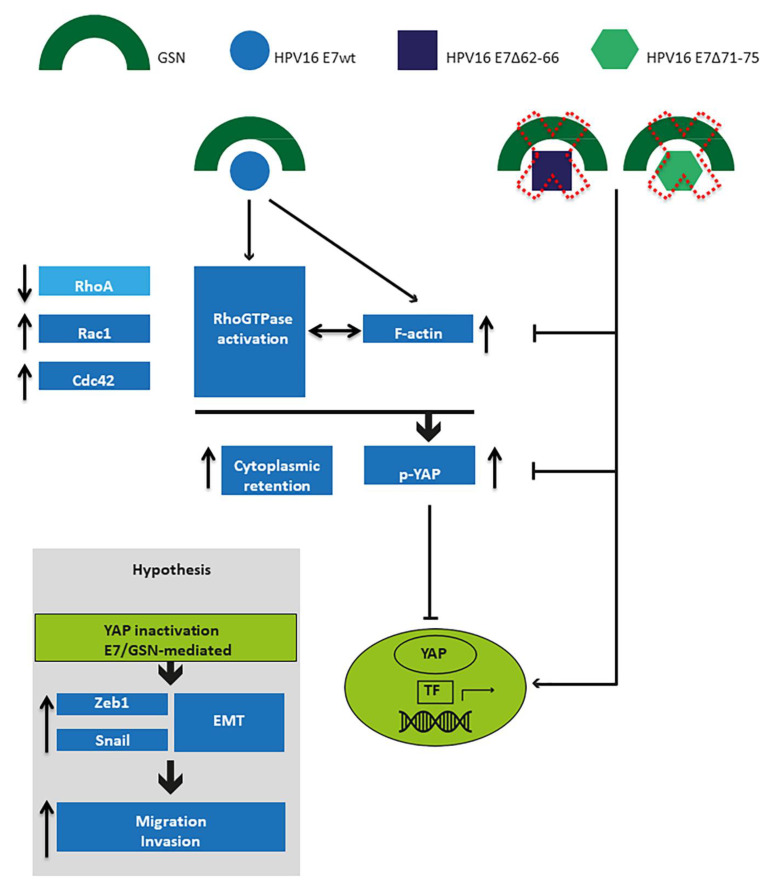
Cartoon illustrating the potential cascade of events due to the physical interaction of E7wt with GSN. In this model, we suggest a crucial role of the molecular association of E7wt with GSN in HPV-positive tumor cell motility and aggressiveness. In this process, the activation of Rac1 and Cdc42 and the increase of polymerization state of actin would be decisive in inducing EMT-related morpho-functional alterations via YAP signaling.

All these data are consistent with the pivotal role of YAP in our experimental model, in which HPV16E7, through its interaction with GNS and the associated morpho-functional alterations of the microfilament system, promotes tumor cell invasion capability and aggressiveness. Figure 7 recapitulates the interplay among E7, GSN and YAP, according to our experimental results.

The requirement of YAP activity for the maintenance of epithelial morphology indicates that YAP could represent a master regulator of the differentiation state [29,40] and, consequently, its inactivation by HPV16E7 via actin remodeling could exploit a crucial role in the “de-differentiation” process elicited by this viral factor in transformed cells, i.e. the partial regression of the epithelial cell HPV16E7-mediated towards a less specialized mesenchymal-like phenotype with a greater propensity to cell motility and invasion. 

Since EMT and associated cell de-differentiation is a pivotal component in cancer progression, our results provide new insights into the oncogenic transformation mechanisms elicited by HPV, mainly by its E7 oncoprotein, in the infected cells, and may suggest a repertoire of targets for therapeutic purposes.

## 5. Conclusions

Our data provide evidence that the E7–GSN interaction promotes the cytoskeleton rearrangement leading to epithelial-mesenchymal transition-linked morphological and transcriptional changes. This process, cytoskeleton remodeling mediated, induces the HIPPO pathway by promoting the cytoplasmic retention of inactive P-YAP. These results suggest that YAP could play a role in the “de-differentiation” process underlying the acquisition of a more aggressive phenotype in HPV16-transformed cells. A deeper comprehension of the multifaceted mechanisms elicited by the HPV infection is vital for providing novel strategies to block the biological and clinical features of virus-related cancers.

## Figures and Tables

**Table 1:** Quantitative cytofluorimetric analyses, restricted to efficiently transfected cells (pAmCyan-positive cells), of AMOT expression level and YAP phosphorylation state in the presence or absence of CytoD. Data are reported as mean ± SD of the median fluorescence intensity values obtained in three independent experiments.

Protein	Mock		E7wt		E7Δ62–66		E7Δ71–75	
	*No CytoD*	*CytoD*	*No CytoD*	*CytoD*	*No CytoD*	*CytoD*	*No CytoD*	*CytoD*
p-YAP	51.4 ± 7.3	53.2 ± 6.1	73.6 ± 7.2	50.4 ± 5.7	39.4 ± 4.5	27.9 ± 3.6	40.0 ± 5.2	30.3 ± 4,0
AMOT1	25.6 ± 4.7	22.6 ± 3.5	23.7 ± 3.9	19.2 ± 2.3	25.1 ± 3.3	20.4 ± 3.1	23.5 ± 3.7	21.3 ± 3.4

## Data Availability

The data supporting reported results are available on request to the corrisponding author and part of them are in the GARRbox repository (https://gbox.garr.it/garrbox/index.php/s/HecFMiV0sMhbnzc).

## Supplementary Materials

The following are available online at https://www.mdpi.com/2072-6694/13/2/353/s1, Figure S1: IVM analysis after double-cell staining with anti-YAP (red) and Hoechst (blue) of cells transfected (green) with pAmCyan empty vector (Mock), E7wt or deletion mutant constructs E7∆62-66, E7∆-71-75, Figure S2: IVM analysis after double-cell staining with anti-p-YAP antibody (red) and Hoechst (blue) of cells transfected (green) with pAmCyan empty vector (Mock), E7wt or deletion mutant constructs E7∆62-66, E7∆71-75, Figure S3: IVM analysis after double-cell staining with anti-AMOT (red) and anti-p-YAP antibody (blue) of cells transfected (green) with pAmCyan empty vector (Mock), E7 wild-type (E7wt) or deletion mutant constructs E7∆62-66 and E7∆71-75, Figure S4: Representative western blot of lysates of C33A cells treated with 0.1% DMSO as vehicle control in EMT markers expression analysis. Uncropped western blot image available in Figure S10, Figure S5. Uncropped western blot images for Figure 1C, Figure S6. Uncropped western blot images for Figure 2A–C, Figure S7. Uncropped western blot images for Figure 3A, Figure S8. Uncropped western blot images for Figure 4A, Figure S9. Uncropped western blot images for Figure 6B, Figure S10. Uncropped western blot images for supplementary Figure S4.
