# Supplementary material for: Physical Interaction between HPV16E7 and the Actin-Binding Protein Gelsolin Regulates Epithelial-Mesenchymal Transition via HIPPO-YAP Axis
Source: Cancers (Basel). 2021 Jan 19;13(2):353. doi: 10.3390/cancers13020353 (PMC7836002; doi:10.3390/cancers13020353)
Supplement: Supplementary file 1 [file cancers-13-00353-s001.pdf]

cancers

MDPI

Supplementary Materials: Physical Interaction Between HPV16E7 and the Actin-Binding Protein Gelsolin Regulates Epithelial-Mesenchymal Transition Via HIPPO-YAP Axis

Paola Matarrese [1,†], Rosa Vona [1,†], Barbara Ascione [1], Marco G. Paggi [2,*] and Anna Maria Mileo [3,*]

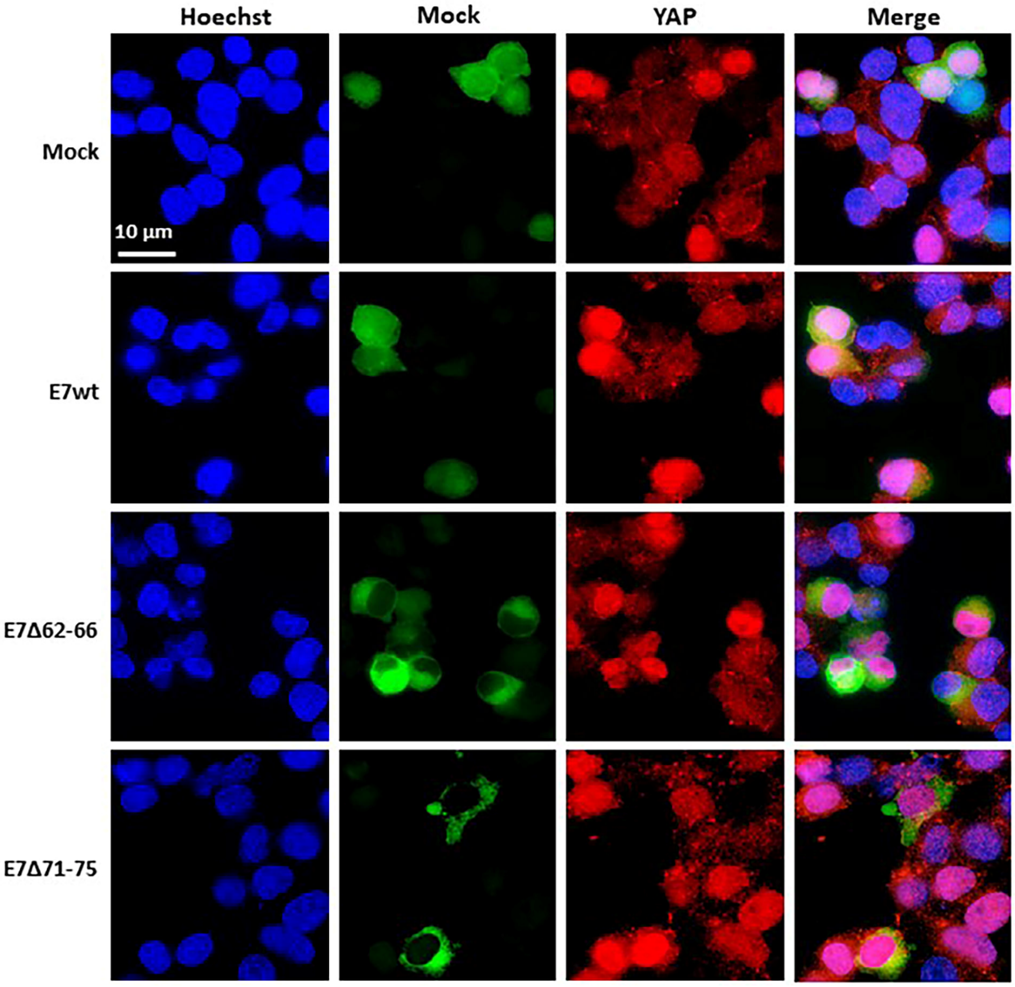

Figure S1. IVM analysis after double-cell staining with anti-YAP (red) and Hoechst (blue) of cells transfected (green) with pAmCyan empty vector (Mock), E7wt or deletion mutant constructs E7Δ62-66, E7Δ-71-75.

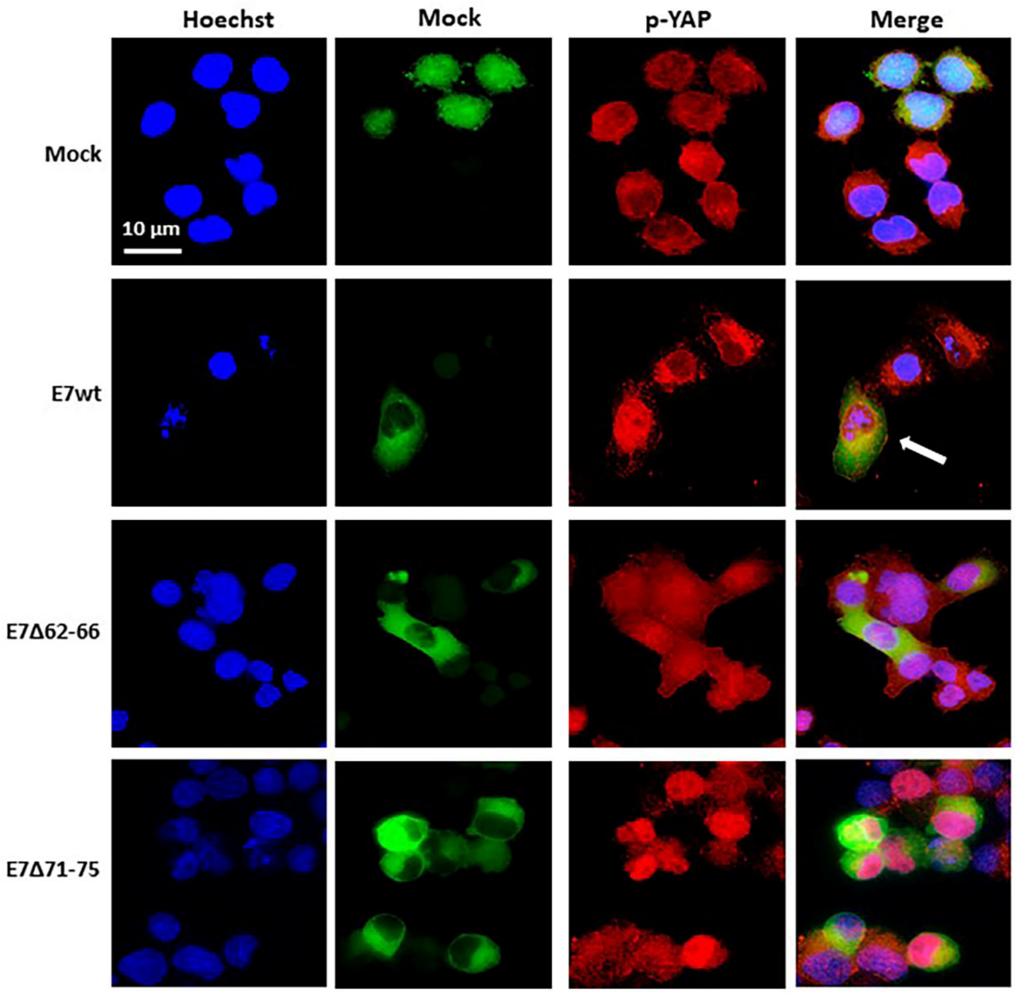

Figure S2. IVM analysis after double-cell staining with anti-p-YAP antibody (red) and Hoechst (blue) of cells transfected (green) with pAmCyan empty vector (Mock), E7wt or deletion mutant constructs E7Δ62-66, E7Δ71-75. Interestingly, p-YAP is found exclusively in the cytoplasm only in E7wt cells (yellow fluorescence, see arrow).

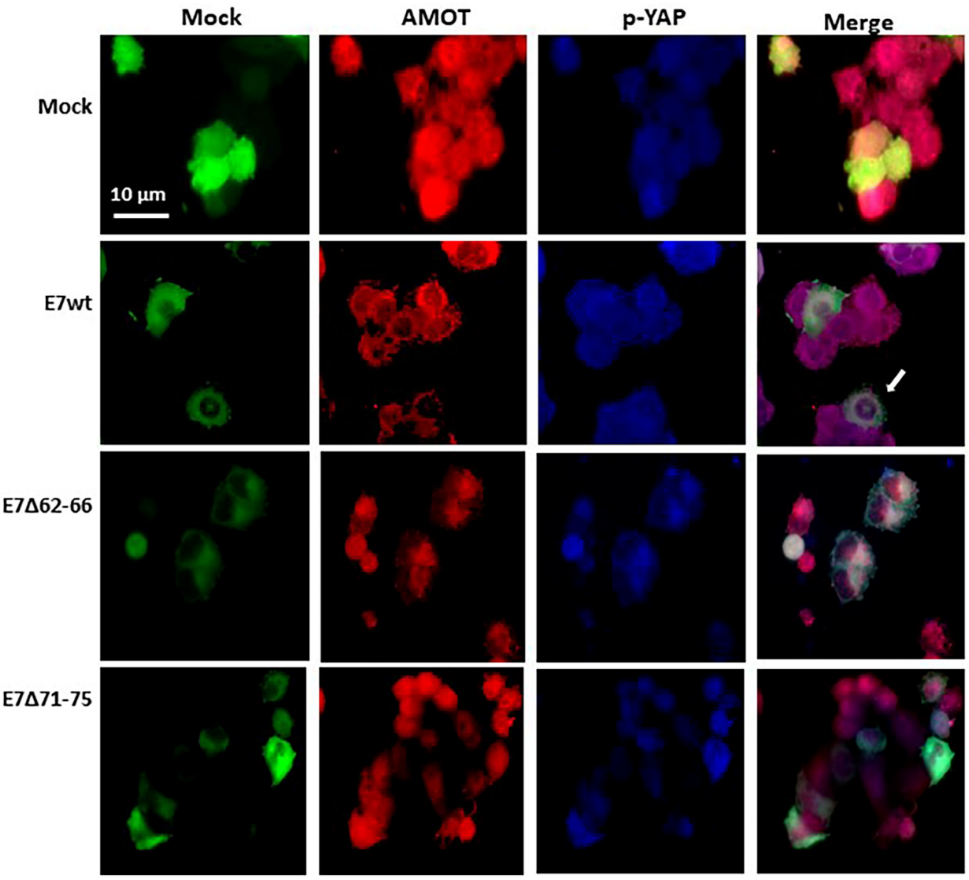

Figure S3. IVM analysis after double-cell staining with anti-AMOT (red) and anti-p-YAP antibody (blue) of cells transfected (green) with pAmCyan empty vector (Mock), E7 wild-type (E7wt) or deletion mutant constructs E7Δ62-66 and E7Δ71-75. To note that p-YAP/AMOT complexes were localized in a cytoplasmic area all around the nucleus in cells expressing HPV16E7wt (arrow). In mock transfected cells, the co-localization of fluorescence signals appeared scattered throughout the cytoplasm, while in cells expressing both E7 mutants it appeared polarized in a restricted cytoplasmic area close to the nucleus.

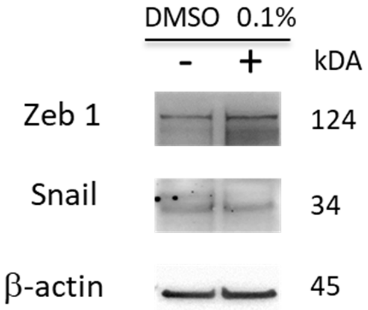

Figure S4. Representative western blot of lysates of C33A cells treated with 0.1% DMSO as vehicle control in EMT markers expression analysis.

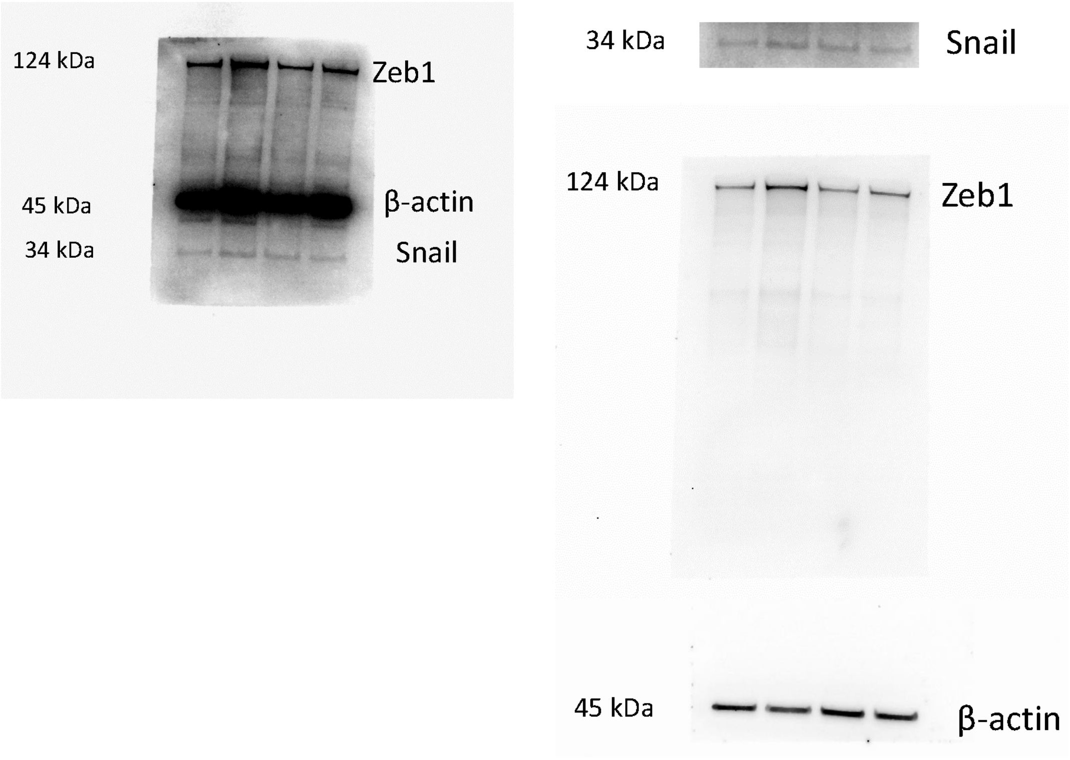

Figure S5. Uncropped western blot images for Figure 1C.

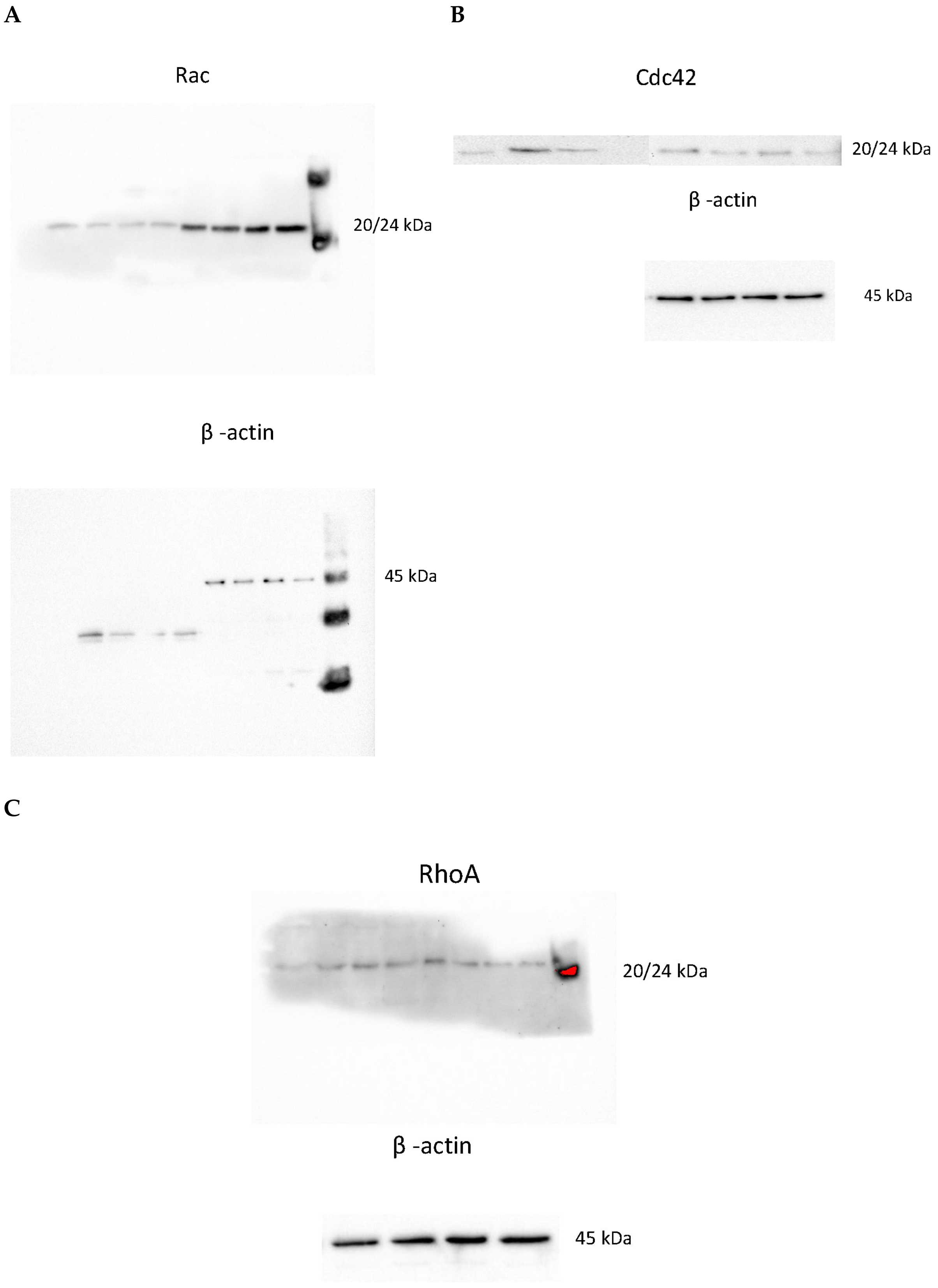

Figure S6. Uncropped western blot images for Figures 2A–C.

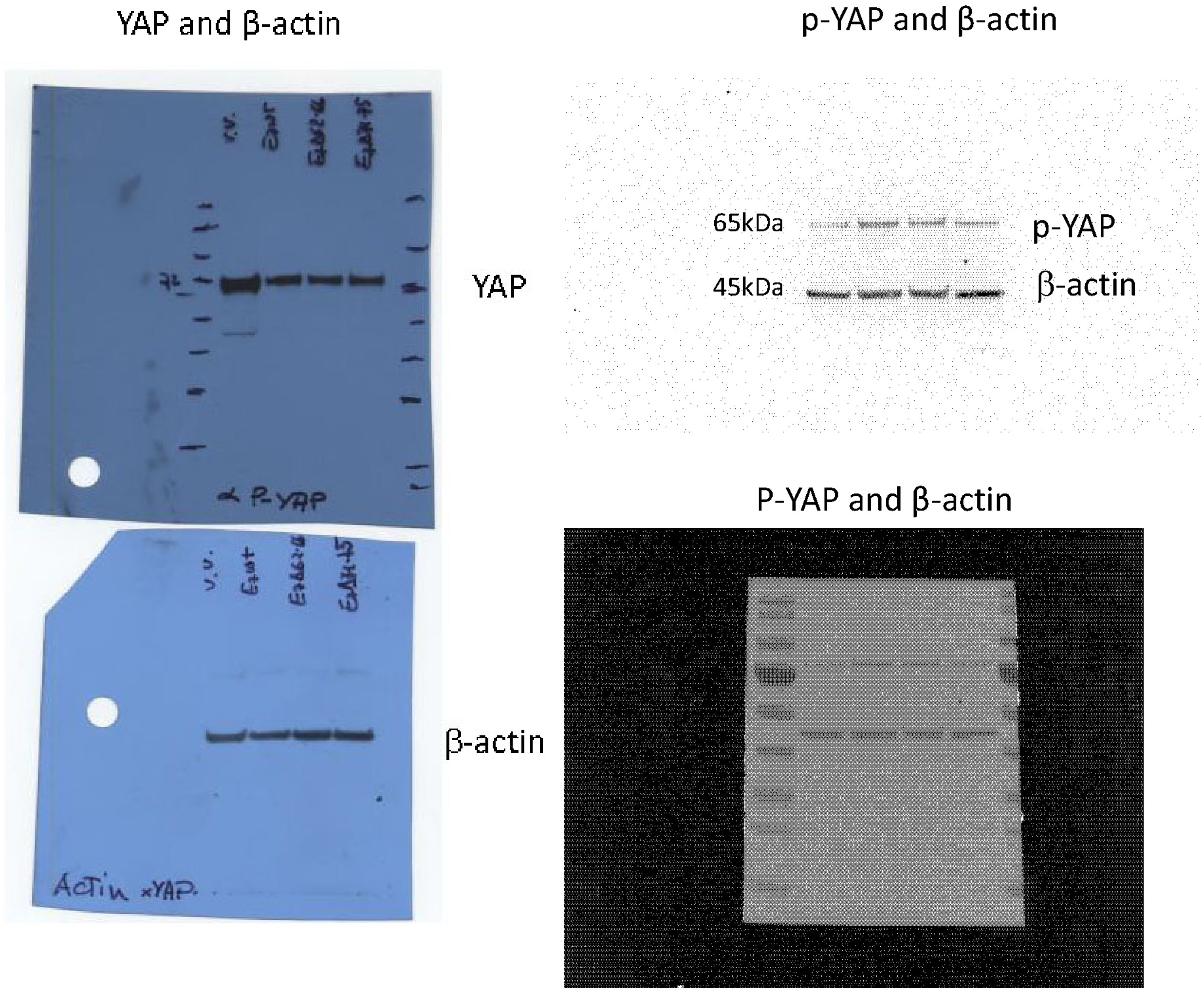

Figure S7. Uncropped western blot images for Figures 3A.

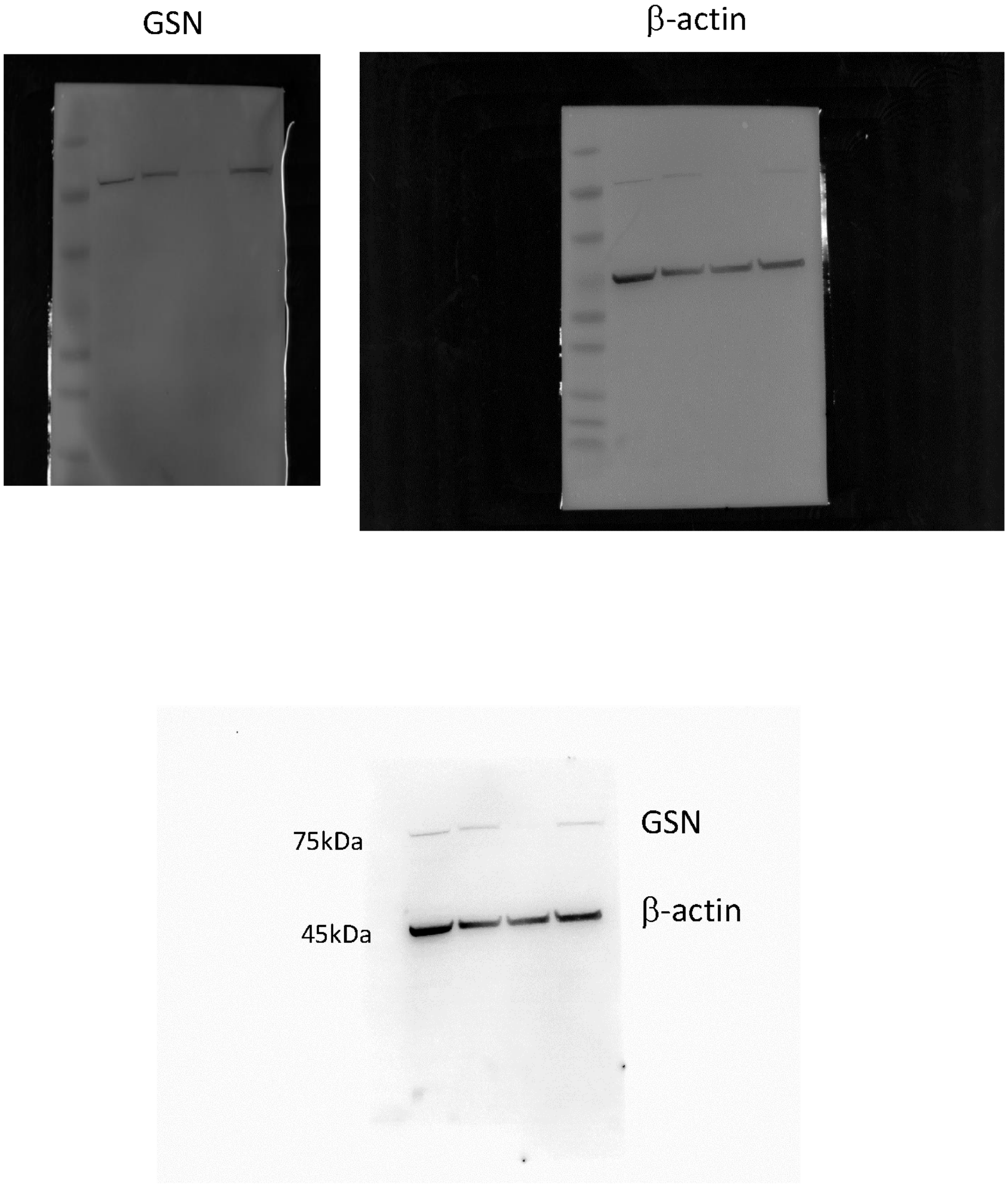

Figure S8. Uncropped western blot images for Figures 4A.

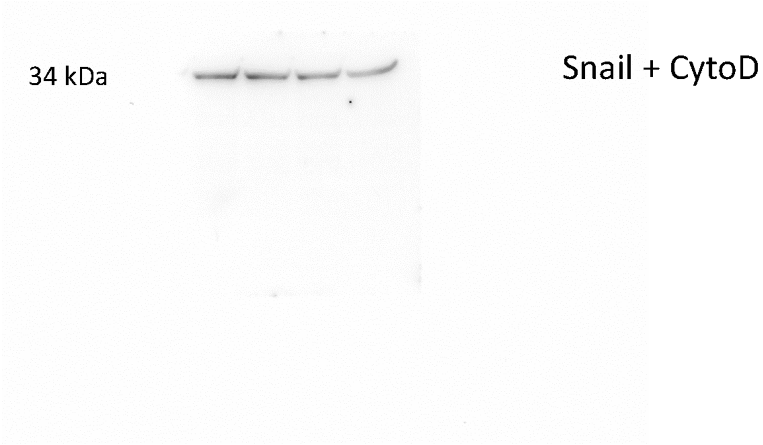

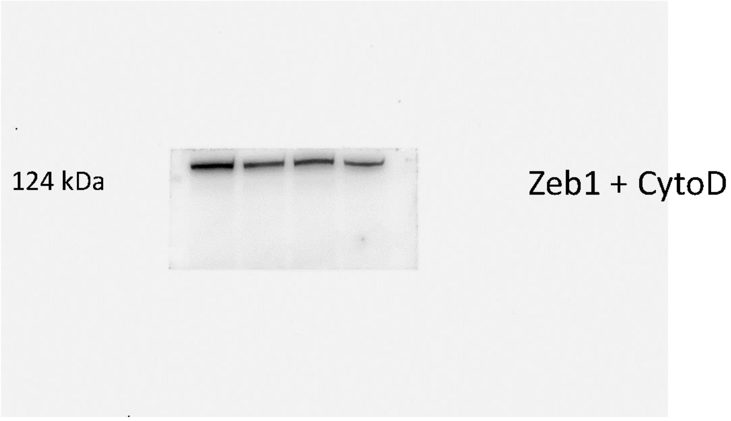

Figure S9. Uncropped western blot images for Figure 6B.

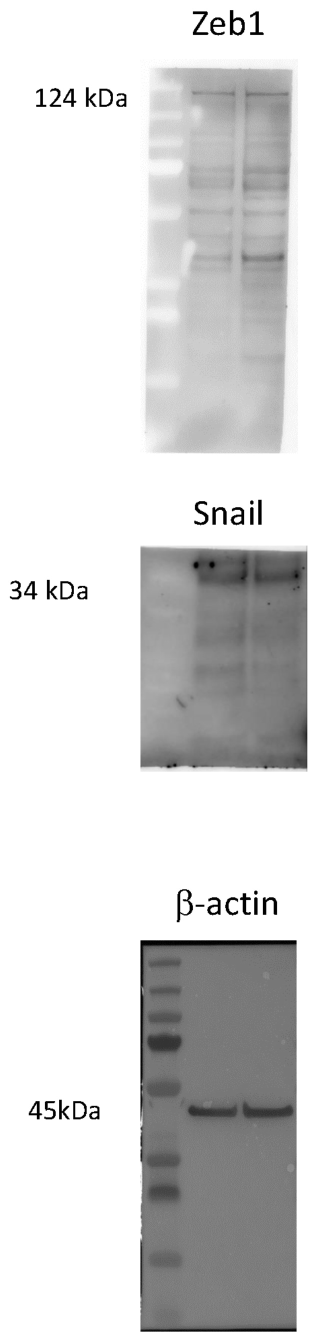

Figure S10. Uncropped western blot images for supplementary Figure S4.
